# Early visual experience and the recognition of basic facial expressions: involvement of the middle temporal and inferior frontal gyri during haptic identification by the early blind

**DOI:** 10.3389/fnhum.2013.00007

**Published:** 2013-01-28

**Authors:** Ryo Kitada, Yuko Okamoto, Akihiro T. Sasaki, Takanori Kochiyama, Motohide Miyahara, Susan J. Lederman, Norihiro Sadato

**Affiliations:** ^1^Department of Physiological Sciences, The Graduate University for Advanced Studies (Sokendai)Okazaki, Japan; ^2^Division of Cerebral Integration, National Institute for Physiological SciencesOkazaki, Japan; ^3^The Hakubi Project, Primate Research Institute, Kyoto UniversityKyoto, Japan; ^4^School of Physical Education, University of OtagoDunedin, New Zealand; ^5^Department of Psychology, Queen's UniversityKingston, ON, Canada; ^6^Biomedical Imaging Research Center, University of FukuiEiheiji, Japan

**Keywords:** blind, facial expression, haptics, supramodal, fMRI, touch, psychophysics

## Abstract

Face perception is critical for social communication. Given its fundamental importance in the course of evolution, the innate neural mechanisms can anticipate the computations necessary for representing faces. However, the effect of visual deprivation on the formation of neural mechanisms that underlie face perception is largely unknown. We previously showed that sighted individuals can recognize basic facial expressions by haptics surprisingly well. Moreover, the inferior frontal gyrus (IFG) and posterior superior temporal sulcus (pSTS) in the sighted subjects are involved in haptic and visual recognition of facial expressions. Here, we conducted both psychophysical and functional magnetic-resonance imaging (fMRI) experiments to determine the nature of the neural representation that subserves the recognition of basic facial expressions in early blind individuals. In a psychophysical experiment, both early blind and sighted subjects haptically identified basic facial expressions at levels well above chance. In the subsequent fMRI experiment, both groups haptically identified facial expressions and shoe types (control). The sighted subjects then completed the same task visually. Within brain regions activated by the visual and haptic identification of facial expressions (relative to that of shoes) in the sighted group, corresponding haptic identification in the early blind activated regions in the inferior frontal and middle temporal gyri. These results suggest that the neural system that underlies the recognition of basic facial expressions develops supramodally even in the absence of early visual experience.

## Introduction

Perception of a face provides a wealth of social information such as identity, age, gender, emotion, gaze direction, and intention. There has been considerable interest in the development of the neural mechanisms that underlie face processing. However, as yet, we do not fully understand well how innate factors and post-natal experience interact to produce these underlying neural mechanisms (Leppänen and Nelson, 2009). In the present study, we have focused our investigation on the effect of early visual deprivation on the acquisition of neural mechanisms that underlie one critical aspect of face perception, namely, the recognition of facial expressions.

Facial expressions play a critical role in understanding one another's mental states. The facial musculature can produce over 40 independent actions, which make possible an extremely large number of expressions. Nevertheless, only a small number of basic facial configurations are spontaneously produced across different cultures (Ekman, 1993; Darwin, 1998). As a consequence, neonates are capable of visually imitating and discriminating among such basic facial expressions (Field et al., 1982). Members of different cultures develop a similar capacity to recognize these facial expressions (Ekman et al., 1969). Such findings raise the possibility that the neural mechanisms that underlie human recognition of basic facial expressions are innately equipped.

As the human face is inherently three-dimensional, geometric change resulting from muscular contraction and relaxation within the face can be detected not only visually, but haptically as well. We have previously shown that with only minimal training sighted humans can haptically recognize basic facial expressions surprisingly effectively (Lederman et al., 2007). Previous electrophysiological and neuroimaging studies have revealed that the distributed neural system including frontal, as well as temporal and occipital cortices are involved in face perception (Ishai, 2008; Tsao et al., 2008a,b; Haxby and Gobbini, 2011). We recently demonstrated that within this neural system, several brain regions involving the inferior frontal gyrus (IFG) and the posterior superior temporal sulcus (pSTS) (including the middle temporal gyrus, MTG) were activated by both visual and haptic identification of basic facial expressions in the sighted individuals (Kitada et al., 2010). This finding suggests that the neural representation responsible for recognizing basic facial expressions may be supramodal, i.e., accessible via haptics, as well as vision. Accordingly, we now ask the following question: are the neural substrates involved in recognizing basic facial expressions present in early blind individuals? If the neural representation underlying the recognition of basic facial expressions is innately acquired, such a network should be accessible to the early blind haptically.

Although the brain network underlying face perception is distributed over the whole brain, previous neuroimaging studies have focused only on the occipito-temporal cortex (Pietrini et al., 2004; Goyal et al., 2006). More critically, neither of these previous studies found a functional architecture responsible for face perception that was common to the early blind and sighted individuals. For instance, Pietrini et al. (2004) showed patterns of activation in the ventral temporal cortex of the early blind that differed for haptically perceived faces versus inanimate objects (i.e., shoes and bottles); however such activation patterns were not observed in the sighted. Therefore, it remains an open question as to whether the common brain network underlying face perception, which is distributed beyond the occipito-temporal cortex, can develop regardless of early visual experience.

The current functional magnetic-resonance imaging (fMRI) study examined the neural representation underlying haptic recognition of basic facial expressions in the absence of early visual experience. Both sighted and early blind subjects haptically identified 3-D facemasks that portrayed three basic facial expressions and 3-D casts of three different shoe types (control). Sighted subjects completed an additional task in which they identified the same 3-D objects visually. We predicted that the early blind group, which is unable to create visual imagery, could still recognize basic facial expressions using their sense of touch. Moreover, if a modality-independent representation of basic facial expressions is present, the neural substrates in the early blind should overlap those of the sighted (using vision or touch). Finally, based on our previous study on the sighted (Kitada et al., 2010), we predicted that such supramodal activation of a cortical network would involve the IFG and pSTS region.

## Materials and methods

### Subjects

A total of 19 early blind and 33 sighted individuals participated in the study (Table 1). None of the subjects reported a history of major medical or neurological illness, such as epilepsy, significant head trauma, or a lifetime history of alcohol dependence. None of the subjects had previously been trained to identify facial expressions haptically. All of the subjects gave written informed consent for participation in the study. The protocol was approved by the local medical ethics committee at the National Institute for Physiological Sciences (Aichi, Japan).

**Table 1:** **Early blind subjects**.

**Subject**	**Sex**	**Handedness**	**Age**	**Onset of total blindness**	**Cause of blindness**	**Residual vision**	**Psychophysics**	**fMRI**
Eb1	M	R	38 years	2 years^*^	Retinoblastoma	No	Yes	Yes
Eb2	F	R	60 years	0 years	Glaucoma	No	Yes	Yes
Eb3	F	R	23 years	0 years	Retinopathy of prematurity	Light perception	Yes	Yes
Eb4	M	R	32 years	3 years^*^	Glaucoma	No	Yes	Yes
Eb5	M	R	37 years	0 years	Retinopathy of prematurity	No	Yes	Yes
Eb6	M	R	23 years	0 years	Amaurosis	Light perception	Yes	Yes
Eb7	M	R	30 years	0 years	Retinopathy of prematurity	Light perception	Yes	Yes
Eb8	F	R	23 years	0 years	Retinopathy of prematurity	Light perception	Yes	Yes
Eb9	M	R	36 years	2 years^*^	Retinoblastoma	No	Yes	Yes
Eb10	F	R	45 years	0 years	Congenital unknown disease	Yes	Yes	Yes
Eb11	F	R	62 years	7 years^*^	Congenital cataract	No	Yes	Yes
Eb12	M	R	28 years	0 years	Microphthalmia	No	Yes	Yes
Eb13	M	L	26 years	0 years	Anophthalmia	No	Yes	No
Eb14	M	R	54 years	0 years	Glaucoma	No	Yes	No
Eb15	M	R	44 years	0 years	Retinopathy of prematurity	No	No	Yes
Eb16	M	R	37 years	0 years	Retinopathy of prematurity	No	No	Yes
Eb17	F	R	58 years	0 years	Congenital unknown disease	Light perception	No	Yes
Eb18	F	R	50 years	0 years	Chorioretinal atrophy	Light perception	No	Yes
Eb19	M	R	29 years	0 years	Retinopathy of prematurity	No	No	Yes

*The “Psychophysics” and “fMRI” columns indicate subject participation in the psychophysical and fMRI experiments, respectively*.

* *Vision of these subjects was substantially reduced well before the clinical diagnosis of total blindness (see the main text)*.

Of the early blind subjects, 15 of 19 reported being totally blind from birth. The other four were severely visually impaired early on, and later became totally blind. More specifically, EB11 suffered from congenital cataracts in both eyes, and reported that she could only see light and color after birth. EB1 and EB9 both suffered from severe retinoblastoma (most likely from birth); the tumor occluded their vision to the extent that both eyes were extirpated. EB4 was diagnosed with glaucoma and reported suffering from severe low vision after birth. None of the early blind subjects could recall ever seeing familiar objects (e.g., faces) after birth.

#### Psychophysical experiment

Fourteen early blind (mean age ± standard deviation [*SD*] = 36.9 ± 13.5 years) and 14 sighted (mean age ± *SD* = 41.3 ± 11.8 years) individuals participated in this experiment (Table 1). Each group consisted of nine males and five females, with 13 right-handed and one left-handed individual as measured by the Edinburgh Handedness Inventory (Oldfield, 1971). There was no significant age difference between the groups (*t*-test, *P* = 0.4).

#### fMRI experiment

Seventeen early blind individuals (10 males and 7 females, mean age ± *SD* = 38.5 ± 12.9 years) and 22 sighted individuals (13 males and 9 females, mean age ± *SD* = 36.5 ± 14.4 years) participated in this experiment (Table 1). We matched the ratio of males to females (59% and 41%, respectively) across the two groups. There was no significant difference in age between the groups (*t*-test, *P* = 0.7). All subjects were right-handed (Oldfield, 1971).

Owing to the challenge of recruiting subjects, 12 early blind and 3 sighted individuals participated in both the psychophysical and fMRI experiments (Table 1). However, the most critical consideration for testing the current hypothesis is the difference between faces and control objects within each group. As shown below, the subjects received extensive preliminary familiarization with both faces and control objects in the fMRI experiment. Thus, brain activation in each group, as revealed by the identification of facial expressions relative to control objects, should not have been affected by greater familiarization with facemasks of early blind as opposed to sighted subjects.

### Stimuli

**Figure 1:**
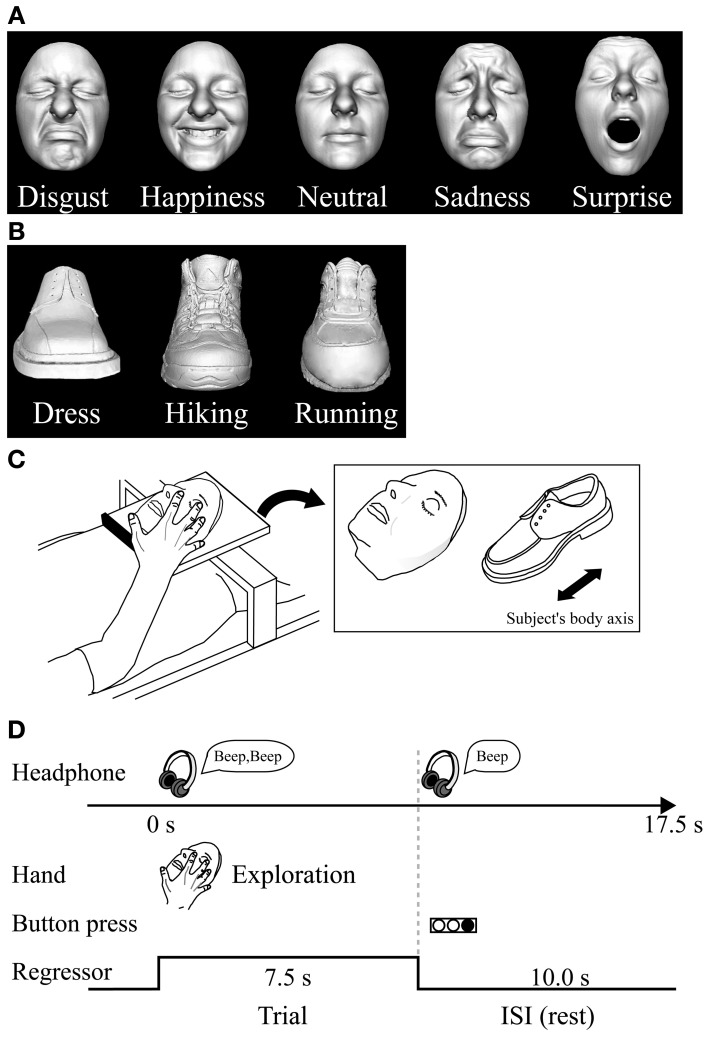
**Haptic task. (A)** Plastic masks, each portraying five different expressions, were utilized as stimuli in the psychophysical experiment. **(B)** The fMRI experiment used plastic masks expressing disgust, happiness, and neutral; three types of shoe (plastic casts) were used as control objects. **(C)** In the fMRI experiment, each exemplar was mounted on a plexiglass table. The orientation of the objects as they were presented on the table is shown on the right. **(D)** Task schedule for the fMRI experiment. Subjects were instructed to explore the object with their right hand immediately after they heard a sound cue though headphones. When another sound cue was presented (after 7.5 s of exploration), the subjects were told to stop. They were instructed to respond immediately by using their left hand to press the button corresponding to the appropriate numeric code for the subordinate-level category presented. Neural activity during the task block was modeled with a box-car function for each object category (i.e., face and shoe). The regressor shown was convolved with a canonical hemodynamic-response function.

We used two different basic-level (Rosch, 1976) classes of objects: plastic casts of faces and shoes (for details of stimuli and their production see Kitada et al., 2010). For the psychophysical experiment, we utilized five basic facial expressions (disgusted, happy, neutral, sad, and surprised faces) (Ekman et al., 1969). We used 10 different facemasks with two actresses producing five facial expressions (Figure 1A).

For the subsequent fMRI experiment, we utilized three subordinate levels of expressions (disgust, neutral, and happiness) and three subordinate levels of shoes (dress, running, and hiking). We prepared three exemplars for each subordinate level (3 exemplars × 3 subordinate levels × 2 basic levels = 18 objects in total; Figures 1A,B). We chose these three facial expressions based on the performance accuracy results in the psychophysical experiment described below. We excluded the surprise faces as stimuli, because their open mouth made the length of the entire face greater than the others, making haptic exploration of the upper face difficult within the scanner where space for manual exploration is highly limited.

### Psychophysical experiment

We tested whether the early blind and sighted subjects were haptically able to recognize facial expressions without corrective feedback. Each subject was blindfolded, wore ear plugs, and sat on a chair in front of a table on which a sheet of acrylic plate was fixed. For each trial, the experimenter placed a facemask at the center of the sheet, facing upwards with the chin located closest to the subject's body. The midline of the facemask was approximately orthogonal to the front plane of the subject. In response to the experimental command of “ready,” the subject placed both hands above the facemask. In response to the command of “go,” the subject was asked to drop both hands down upon the facemask and to explore it. The subject was instructed to indicate orally which of the five expressions each facemask portrayed, as accurately as possible and within 60 s. Response time was measured with a stopwatch. The task design included eight blocks of trials, each comprising the facemasks of one actress expressing five different expressions. The faces of the two actresses were presented in separate blocks, in alternating order (5 expressions × 2 actresses × 4 blocks = 40 trials). The facemasks within each block were presented in a pseudo-randomized order. The first two blocks presenting all of the expressions portrayed by the two actresses served as practice trials (10 trials in total), in order to familiarize the subjects with the task. However, the subjects did not receive corrective feedback during either the practice or test trials.

### fMRI experiment

After the psychophysical experiment, we conducted an fMRI experiment to test the hypothesis that common neural correlates underlie identification of facial expressions in both early blind and sighted individuals.

#### Data acquisition

fMRI was performed using a 3T Siemens Allegra whole-head MRI system (Siemens, Erlangen, Germany). Standard sequence parameters were used to obtain the functional images as follows: gradient-echo echo-planar imaging (EPI); repetition time (TR) = 2500 ms; echo time (TE) = 30 ms; flip angle = 80°; 39 axial slices of 3-mm thickness with a 17% slice gap; field of view = 192 × 192 mm; and in-plane resolution = 3.0 × 3.0 mm. A T1-weighted high-resolution anatomical image was obtained from each participant (voxel size = 0.9 × 0.9 × 1 mm) between the runs obtaining functional data.

#### Haptic-identification task

This task was designed to examine the cortical networks involved in haptic identification of facial expressions. The sighted subjects were not allowed to see the objects until the final run of the haptic object-identification task was completed.

***Haptic stimulus presentation.*** The subjects lay supine on a bed with their eyes closed, wearing MRI-compatible headphones (Hitachi Advanced Systems, Yokohama, Japan), and were instructed to relax. We followed the same procedure as that used in a previous fMRI study to present objects haptically (Kitada et al., 2010). The right hand was used to explore the stimuli, while the left arm was extended along the side of the subject's body and the left hand held a response pad (Figure 1C).

Each subject completed four runs of the haptic object-identification task (146 volumes per run, 365 s). A single run consisted of a 315-s task period preceded by 32.5 s and followed by 17.5-s rest periods. Each of the 18 exemplars was presented once during the task period, for 7.5 s (Figure 1C). This trial duration was based on results from our previous study (Kitada et al., 2010), where 7.5 s was sufficient for the subject to identify three facial expressions after a short period of training, which is explained in the next section. The presentation of objects alternated with 10.0 s inter-stimulus intervals (ISI), during which subjects identified the previous object with a key press (17.5 s × 18 exemplars = 315 s in total). The order in which the objects were presented in a single run was pseudo-randomized using a genetic algorithm that maximized the estimation efficiency for the tested contrasts (Wager and Nichols, 2003). Presentation software (Neurobehavioral Systems Inc., Albany, CA) was used to present auditory cues to the subject via headphones and visual cues to the experimenter during the haptic-identification task; it was also used to present the face and shoe stimuli during the subsequent visual-identification task.

***Task.*** Before the fMRI experiment, subjects were blindfolded and trained to identify the stimulus objects until they felt comfortable performing the task. Unlike the psychophysical experiment, training with corrective feedback was necessary to minimize activation due to a possible difference in task difficulty between the identification of faces and shoes. During training, subjects were asked to identify objects at the subordinate-level of categorization (i.e., the specific facial expression or shoe type). The same set of objects was used during training and in the fMRI experiment. We confirmed that the hand movements used to explore objects were comparable across object categories in terms of exploratory procedures (Lederman and Klatzky, 1987). Training took less than 30 min.

In each trial, subjects were instructed to start exploring the object as soon as they heard a brief sound cue (Figure 1D). Another cue was presented 7.5 s after the trial was initiated. Subjects were asked to report the subordinate-level category immediately by pressing one of the three buttons. To match the sensorimotor components between object classes, subjects were instructed to continue exploring the object to confirm their answer even if they had identified it within 7.5 s.

#### Visual-identification task

After completion of the haptic task, sighted subjects participated in a visual-identification task to enable us to localize the brain regions involved in visual identification of facial expressions.

***Visual stimulus presentation.*** A monochromatic image of each exemplar was used for the task (Figures 1A,B, see Kitada et al., 2010 for production of stimuli). The differences in size and perceived brightness of these images were minimized using photo-editing software (Photoshop, Adobe Systems, San Jose, CA). The subjects fixated on a white cross on the screen, which they viewed through a mirror attached to the head coil. Stimuli were back-projected via a liquid crystal display (LCD) projector (LT 265, NEC Viewtechnology, Tokyo, Japan) onto a translucent screen located at the rear of the scanner. The stimuli and the white fixation cross subtended visual angles of approximately 8.5 and 0.9°, respectively. The right hand did not touch any object during the visual task, while the left hand held the response pad.

**Figure 2:**
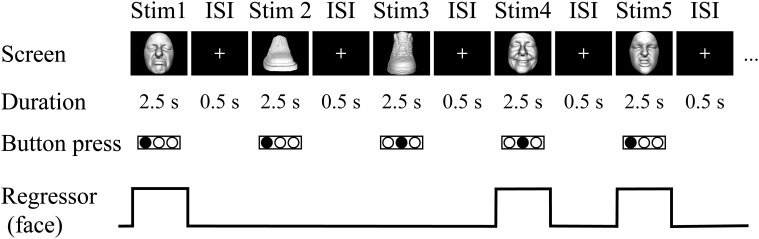
**Visual task.** In the fMRI experiment, sighted subjects were instructed to identify the three subordinate levels for both object categories by pressing one of three buttons on the response pad with their left hand. Neural activity during the trials was modeled with a box-car function for each object category (i.e., face and shoe).

***Task.*** The visual-identification task was the same as the haptic task in that the subject identified the facemasks and shoes presented in a pseudo-randomized order. More specifically, the visual task consisted of two runs (131 volumes per run, 327.5 s). A single run consisted of a 270-s task period that was preceded by a 32.5-s fixation (rest) period and followed by a 25-s fixation (rest) period. During the task period, each of the 18 exemplars was presented five times. Each image appeared for 2.5 s with an ISI of 0.5 s (3.0 s × 90 images = 270 s, Figure 2). The order of object presentation within a single run was pseudo-randomized using the same algorithm as in the haptic-identification task. Subjects were asked to identify the facial expressions or shoe types by pressing one of the three buttons. The fMRI experiment was conducted after approximately 10 min of training. The same set of stimuli was used during the training and the fMRI experiment.

### Data processing

Image processing and statistical analyses were performed using the Statistical Parametric Mapping package (SPM8, Friston et al., 2007). The first five volumes of each fMRI run were discarded to allow the MR signal to reach a state of equilibrium. Functional images from each run were realigned to the first data scan to correct for motion. All functional images and T1-weighted anatomical images were then co-registered to the first scan of the haptic-identification task. Each co-registered T1-weighted anatomical image was normalized to a standard T1 template image (ICBM 152), which defined the Montreal Neurological Institute (MNI) space. The parameters from this normalization process were then applied to each functional image. The normalized functional images were filtered using a Gaussian kernel of 8 mm full width at half maximum (FWHM) in the *x*, *y*, and *z* axes.

### Statistical analysis

Linear contrasts between conditions in the haptic and visual tasks were calculated for individual subjects, and incorporated into a random-effects model to make inferences at a population level (Holmes and Friston, 1998).

#### Initial individual analysis

For each subject in each group, a design matrix comprising the four runs of the haptic-identification task was prepared. Another design matrix, which included the two runs of the visual-identification task, was prepared separately for the sighted subjects. We fitted a general linear model to the fMRI data for each subject (Friston et al., 1994; Worsley and Friston, 1995). Neural activity during both tasks was modeled with box-car functions convolved with the canonical hemodynamic-response function. Each run included two task-related regressors, one for each object category (faces and shoes). As we previously confirmed that visual and haptic recognition of our stimuli yield no expression-specific activation in the sighted (Kitada et al., 2010), we averaged activation for these facial expressions as a single regressor. The time series for each voxel was high-pass filtered at 1/128 Hz. Assuming a first-order autoregressive model, the serial autocorrelation was estimated from the pooled active voxels with the restricted maximum likelihood (ReML) procedure, and was used to whiten the data and design matrix (Friston et al., 2002). Motion-related artifacts were minimized by incorporating six parameters (three displacements and three rotations) from the rigid-body realignment stage into each model. Three additional regressors, describing intensities in white matter, cerebrospinal fluid, and residual compartments (outside the brain and skull), were added to the model to account for image-intensity shifts attributable to the movement of the hand within the main magnetic field of the scanner (Grol et al., 2007). The estimates for each condition were evaluated using linear contrasts.

#### Subsequent random-effects group analysis

Contrast images from the individual analyses were used for the group analysis, with between-subjects variance modeled as a random factor. The contrast images obtained from the individual analyses represent the normalized task-related increment of the MR signal of each subject. We employed a flexible factorial design to construct a single design matrix involving the visual and haptic conditions of the sighted individuals, and the haptic conditions of the blind individuals. Conditions for sighted and blind individuals were modeled as separate between-subject (independent) levels, whereas conditions within each group were modeled as within-subject (dependent) levels. The estimates for these conditions were compared using linear contrasts. The resulting set of voxel values for each contrast constituted the SPM{t}. The SPM{t} was transformed into normal distribution units (SPM{z}). The threshold for the SPM{z} was set at *Z* > 2.58 (equivalent to *P* < 0.005 uncorrected). The statistical threshold for the spatial extent test on the clusters was set at *P* < 0.05 and corrected for multiple comparisons over the search volume (Friston et al., 1996). Brain regions were anatomically defined and labeled according to a probabilistic atlas (Shattuck et al., 2008). We evaluated the predefined contrasts described below.

***The sighted group.*** The present study was designed to test whether the IFG and pSTS region, which are active during recognition of facial expressions in the sighted individuals, are also involved in haptic identification of facial expressions in the early blind individuals. We initially confirmed brain regions involved in identification of facial expressions in the sighted individuals, regardless of sensory modality (Kitada et al., 2010). More specifically, we compared the identification of facial expressions with the identification of shoes in each sensory modality (*V*_FE_ − *V*_S_ for vision and *H*_FE_ − *H*_S_ for haptics). Subsequently, we conducted a conjunction analysis to identify common brain regions between *V*_FE_ − *V*_S_ and *H*_FE_ − *H*_S_ (conjunction-null hypothesis; Friston et al., 2005; Nichols et al., 2005). This conjunction approach should eliminate modality-specific interactions such as motoric differences in haptic exploration between faces and control objects. As we previously observed activation in the left IFG and pSTS region (Kitada et al., 2010), the search volume for activation in the left IFG and pSTS region was limited to each of the anatomically defined regions (26,784 mm^3^ for the left IFG and 30,008 mm^3^ for the left posterior superior temporal gyrus and MTG, Shattuck et al., 2008). The search volume for activation in the brain regions excluding the left IFG and pSTS region was the whole brain.

***The early blind group.*** We evaluated the haptic identification of facial expressions relative to shoes (*H*_FE_ − *H*_S_) in the early blind group. We hypothesized *a priori* that *H*_FE_ − *H*_S_ in the early blind activates the left IFG and pSTS, which are also activated by both *V*_FE_ − *V*_S_ and *H*_FE_ − *H*_S_ in the sighted. Therefore, the search volume for activation in the left IFG and pSTS region was limited to the brain regions depicted by a conjunction analysis of *V*_FE_ − *V*_S_ and *H*_FE_ − *H*_S_ in the sighted (1376 mm^3^ for the left IFG and 2416 mm^3^ for the left pSTS region). The search volume for activation in the brain regions excluding the left IFG and pSTS region was the whole brain.

After depicting brain regions revealed by *H*_FE_ − *H*_S_ in the early blind group, we extracted the contrast estimate (i.e., activity during recognition of facial expressions relative to that of control objects) from an 8-mm diameter sphere centered on the peak coordinate of each region (8 mm is the size of the spatial smoothing kernel applied to these data). In order to confirm the negligible effect of early visual experience, we conducted the following two analyses. First, we examined the correlation between the contrast estimate and the age at onset of total blindness in all 17 early blind subjects. Second, we tested if the contrast estimate in the 14 congenitally blind subjects was greater than zero.

## Results

### Psychophysical experiment

#### Performance accuracy

**Figure 3:**
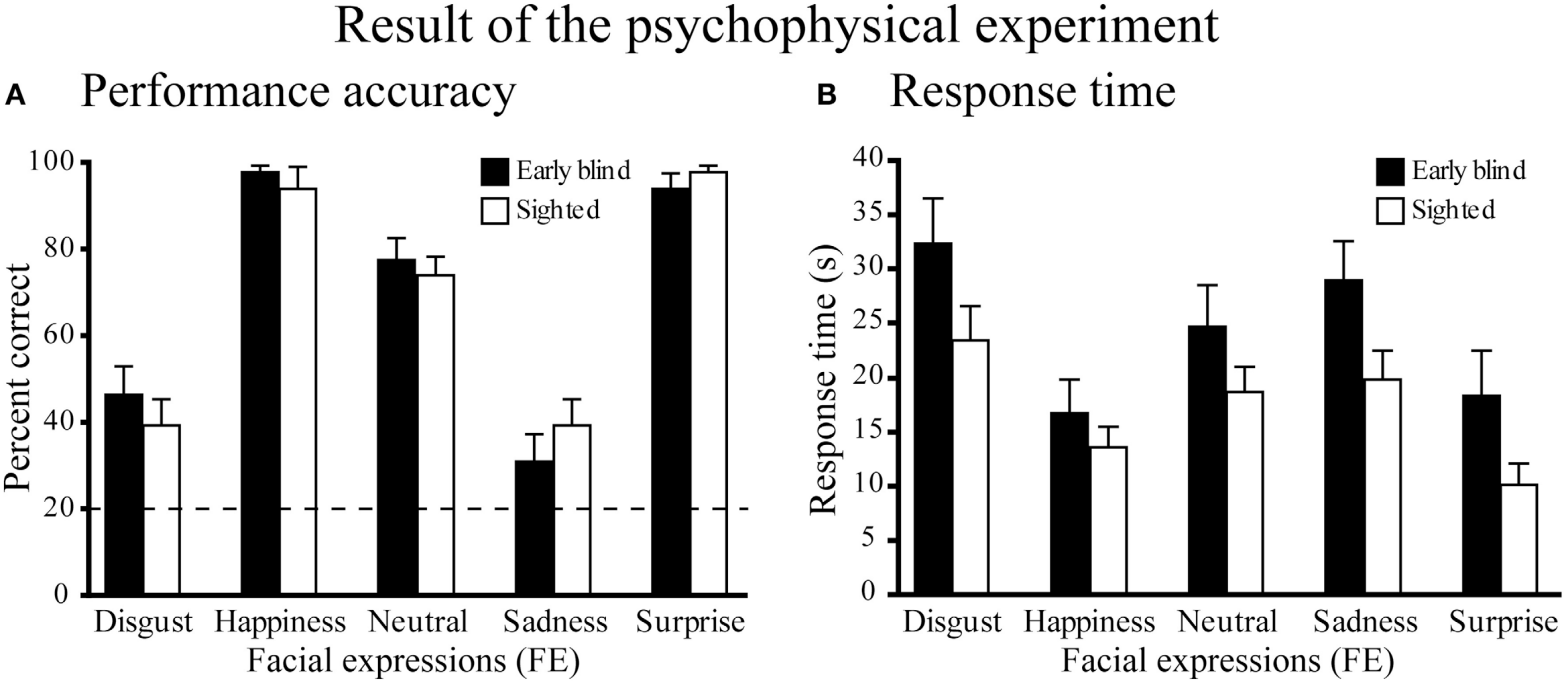
**Results of the psychophysical experiment. (A)** Performance accuracy. Dotted line indicates the level of chance performance (20%). A two-way ANOVA (5 expressions × 2 groups) on the percent correct scores produced a significant main effect of expressions (*P* < 0.001), but neither a significant main effect of group nor a significant interaction between expression and group (*P*-values > 0.4). One-sample *t*-tests on the percent correct scores showed that the accuracy for each facial expression was significantly above chance (*P*-values < 0.01). **(B)** Response time. The same two-way ANOVA on the response time produced a significant main effect of expression (*P* < 0.001), but neither the main effect of group nor the interaction between expression and group were significant (*P*-values > 0.1). Data are presented as the mean ± SEM of 14 subjects for each group.

Performance accuracy across the five expressions was similar between the two groups (Figure 3A). A Two-Way Mixed ANOVA (5 expressions × 2 groups) on the percent correct scores produced a significant main effect of expressions [*F*_(4, 104)_ = 77.6, *P* < 0.001], but neither a significant main effect of group nor a significant interaction between expression and group (both *P*-values >0.4). *Post-hoc* pairwise comparisons (with a Sidak-Bonferroni correction) revealed that the percent correct scores for happiness and surprise ratings were significantly higher than those for the other expressions (*P*-values <0.01). Accuracy for the neutral face was significantly higher than for both disgusted and sad faces (*P*-values <0.001). There were no significant differences between the scores of happy and surprised faces, or between the scores of disgusted and sad faces (*P*-values >0.8). One-sample *t*-tests showed that the accuracy scores for each facial expression were significantly above chance (20%, *P*-values <0.01).

#### Response times

Figure 3B shows the response times for each facial expression across the two groups. A Two-Way ANOVA (5 facial expressions × 2 groups) produced a significant main effect of expression [*F*_(4, 104)_ = 33.5, *P* < 0.001], but neither the main effect of group nor the interaction between expression and group were significant (*P*-values >0.1). *Post-hoc* pairwise comparisons (with a Sidak-Bonferroni correction) revealed significantly shorter response times for happiness and surprise compared with the other facial expressions (*P*-values <0.01). The response time for the neutral face was significantly shorter than those for disgusted face and sad face (*P*-values <0.05) and the response time for sad face was significantly shorter than that for disgusted face (*P*-values <0.05). There was no significant difference between the response times for happy and surprised faces (*P* = 1.0).

#### Does the onset of total blindness affect behavioral performance?

Four early blind subjects had severe low vision at birth before becoming totally blind (Table 1). In order to test if such post-natal visual experience affected the behavioral results among the early blind subjects, we conducted the two additional analyses. First, after excluding the four early blind subjects who became totally blind after birth, we conducted the same Two-Way ANOVA (5 expressions × 2 groups) separately on performance accuracy and response time. In keeping with the previous analyses that used all 14 early blind subjects, neither the main effects of group (*P*-values >0.1 for performance accuracy and response time) nor the interactions of expression and group (*P*-values >0.08) was statistically significant.

Second, we examined the relationship between the age at onset of total blindness in the early blind subjects and their behavioral performance. We calculated mean performance accuracy and mean response time across expressions for each early blind subject. Neither mean performance accuracy nor mean response time was significantly correlated with the age at onset of total blindness (absolute value *r* values <0.1).

### fMRI experiment

#### Task performance

**Figure 4:**
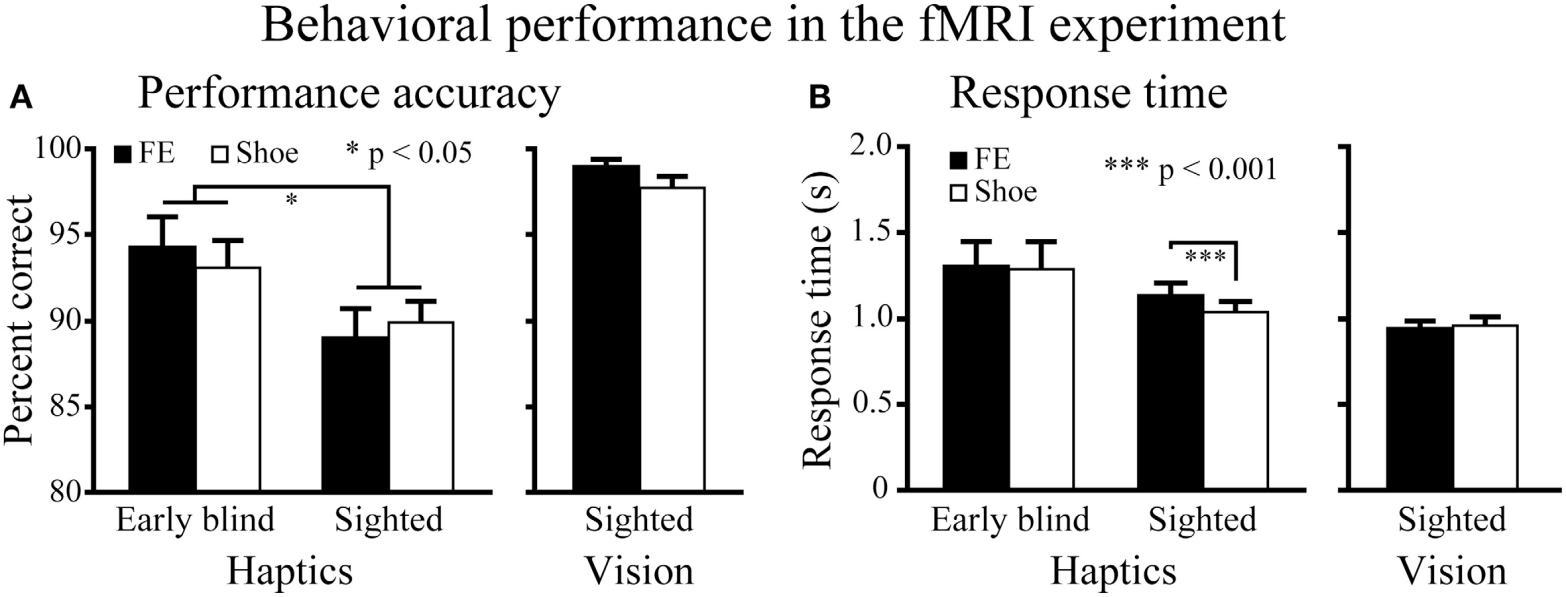
**Behavioral results for the fMRI experiment. (A)** Performance accuracy. Asterisks between the two groups indicate a significant main effect of group (ANOVA). **(B)** Response time. Asterisks between the conditions indicate a significant difference between faces and shoes in the sighted group (paired *t*-test). FE indicates facial expressions. Data are presented as the mean ± SEM of 17 early blind and 22 sighted subjects.

***Haptic object-identification task.*** Performance accuracy was similar for the faces and control objects (shoes; Figure 4). A Two-Way ANOVA (2 object categories × 2 groups) on accuracy scores produced a significant main effect of group [*F*_(1, 37)_ = 4.9, *P* < 0.05], with early blind subjects being more accurate than sighted subjects. More critical to our hypothesis, however, neither the main effect of object category nor the object category by group interaction was significant (*P*-values >0.3).

The same ANOVA performed on the response times revealed a significant main effect of object category: faces produced significantly longer response times than control objects [*F*_(1, 37)_ = 6.6, *P* < 0.05]. In addition, the interaction between object category and group showed a trend toward significance [*F*_(1, 37)_ = 3.9, *P* = 0.055].

A paired *t*-test on response times comparing faces and control objects showed no significant difference in the early blind group (*P* = 0.7), whereas the same test showed that faces produced significantly longer response times than control objects in the sighted group [*t*_(21)_ = 4.16, *P* < 0.001]. However, the difference (104 ms) represented only 1.4% of the total duration of haptic exploration (7500 ms), and within the context of the present study was thus negligible for purposes of evaluating our current hypothesis.

***Visual object-identification task.*** Performance accuracy and response times were comparable for faces and shoes (Figure 4). We found no significant differences in either accuracy or response times between faces and shoes (paired *t*-tests, *P*-values >0.07).

#### Random-effects group analysis

We initially confirmed the brain regions in the sighted group activated by identification of facial expressions relative to shoes, regardless of sensory modality. Subsequently, we tested if the brain regions activated in the sighted group was also observed by haptic identification of facial expressions (relative to shoes) in the early blind group.

**Figure 5:**
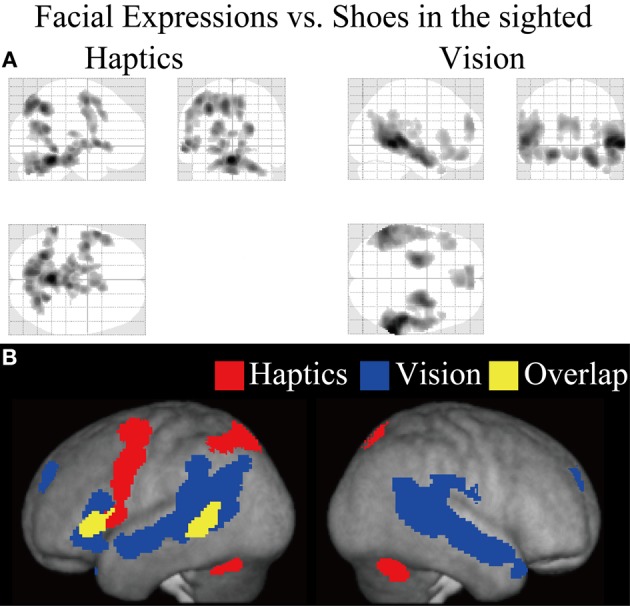
**Statistical parametric mappings (SPM) of the average neural activity within the sighted group during visual and haptic identification of facial expressions compared with shoes (*V*_FE_ − *V*_S_ for vision and *H*_FE_ − *H*_S_ for haptics).** The size of activation was thresholded at *P* < 0.05, corrected for multiple comparisons, when the height threshold was set at *Z* > 2.58. **(A)** The three-dimensional information was collapsed into two-dimensional sagittal, coronal, and transverse images. **(B)** The activation patterns during identification of facial expressions relative to shoes were superimposed on a surface-rendered T1-weighted high-resolution MRI averaged across the subjects. Regions in yellow were activated by both vision and haptics (conjunction analysis).

***Sighted group.*** We evaluated the contrast of the visual identification of facial expressions relative to shoes (*V*_FE_ − *V*_S_, Table 2 and Figure 5). This contrast showed regions of significant activation bilaterally in the superior temporal gyrus, MTG, supramarginal gyrus, superior frontal gyrus, and amygdala. Moreover, significant activation was also observed in the right inferior temporal gyrus, right insula, right putamen, left angular gyrus, left hippocampus, left middle frontal gyrus, left cingulate gyrus, left lateral orbitofrontal gyrus, and left IFG. In addition, we also observed activation in the right middle fusiform gyrus (*x* = 44, *y* = −50, *z* = −24, *Z* value = 4.14); however, the size of these clusters did not exceed the cluster threshold.

**Table 2:** **Group analyses of the brain regions in the sighted subjects**.

**Spatial extent test**		**MNI coordinate**			***Z*-value**	**Hemisphere**	**Anatomical region**
**Cluster size (mm^3^)**	***P*-values**	***x***	***y***	***z***			
**VISUALLY PRESENTED FACIAL EXPRESSIONS RELATIVE TO SHOES (*V*_FE_ − *V*_S_)**							
41952	<0.001	32	0	−22	6.39	R	Inferior temporal gyrus
		64	−44	2	Inf	R	Middle temporal gyrus
		52	−34	6	7.68	R	Superior temporal gyrus
		66	−32	26	3.61	R	Supramarginal gyrus
		36	−16	14	3.74	R	Insula
		28	−6	−16	6.70	R	Amygdala^a^
		34	−10	0	3.85	R	Putamen
31600	<0.001	−58	−52	6	6.98	L	Middle temporal gyrus
		−60	−44	−2	5.64	L	Middle temporal gyrus
		−58	−24	−2	4.82	L	Superior temporal gyrus
		−46	−54	14	5.86	L	Angular gyrus
		−62	−44	22	4.74	L	Supramarginal gyrus
6928	<0.01	−24	−12	−12	6.58	L	Hippocampus/Amygdala^b^
6016	<0.01	−10	54	24	4.78	L	Superior frontal gyrus
		8	56	22	4.70	R	Superior frontal gyrus
		−14	54	8	3.23	L	Middle frontal gyrus
4264	<0.05	4	44	−14	4.68	R	Superior frontal gyrus
		−8	30	−4	3.01	L	Cingulate gyrus
4904	<0.05	−42	32	−8	4.23	L	Lateral orbitofrontal gyrus
		−52	22	6	4.04	L	Inferior frontal gyrus^c^
		−40	26	−14	3.92	L	Lateral orbitofrontal gyrus
**HAPTICALLY PRESENTED FACIAL EXPRESSIONS RELATIVE TO SHOES (*H*_FE_ − *H*_S_)**							
2608	<0.01^*^	−60	−42	−4	4.53	L	Middle temporal gyrus
12984	<0.001	−48	24	0	4.2	L	Inferior frontal gyrus^d^
		−28	0	56	3.75	L	Middle frontal gyrus
		−42	−2	56	4.59	L	Precentral gyrus^e^
4616	<0.05	−18	−66	26	4.26	L	Superior parietal lobule
		−28	−66	8	2.83	L	Lingual gyrus^f^
14144	<0.001	−30	−56	46	5.07	L	Superior parietal lobule
		10	−72	52	4.22	R	Precuneus
4272	<0.05	4	10	54	4	R	Superior frontal gyrus
41960	<0.001	6	−24	−16	4.79	R	Brainstem
		−10	−22	−18	4.18	L	Brainstem
		−2	−46	−18	5.79	L	Cerebellum
		−16	10	2	3.34	L	Putamen
		−16	14	4	3.29	L	Caudate nucleus
**CONJUNCTION OF *V*_FE_ − *V*_S_ and *H*_FE_ − *H*_S_**							
2416	<0.001^*^	−60	−42	−4	4.53	L	Middle temporal gyrus
1376	<0.05^*^	−50	24	2	3.85	L	Inferior frontal gyrus^g^

*The threshold size of activation was P < 0.05, corrected for multiple comparisons, when the height threshold was set at Z > 2.58. x, y, and z are stereotaxic coordinates (mm)*.

*Inf, Z value > 8.0; Hem, hemisphere; R, right; L, left*.

* *The search volume for activation within the left IFG and pSTS region (including the middle temporal gyrus) was limited to each of the anatomically defined regions (Shattuck et al., 2008)*.

^a-g^ Probability values on cytoarchitectonic maps (Amunts et al., 1999, 2000, 2005; Geyer, 2003) are as follows:

a 90% for the amygdala;

b 50% for the hippocampus and 40% for the amygdala;

c 40% for area 44 and 40% for area 45;

d 20% for area 44 and 30% for area 45;

e 40% for area 6;

f 30% for area 17;

g *20% for area 44 and 40% for area 45*.

We evaluated the contrast of haptic identification of facial expressions relative to shoes (*H*_FE_ − *H*_S_, Table 2 and Figure 5). This contrast showed regions of significant activation in the left IFG and left MTG. In addition, we observed significant activation in the left middle frontal gyrus, left precentral gyrus, left superior parietal lobule, left lingual gyrus, right precuneus, right superior frontal gyrus, left cerebellum, left putamen, left caudate nucleus, and bilateral brainstem. According to probabilistic cytoarchitectonic maps (Eickhoff et al., 2005), the activation peak in the left precentral gyrus was located within area 6 (with 40% probability; Geyer, 2003) rather than in the primary motor cortex (0% probability of being located in area 4; Geyer et al., 1996). Consistent with Kitada et al. (2010), we observed no activation in the fusiform gyrus in the random-effect group analysis.

Finally, the conjunction analysis of *V*_FE_ − *V*_S_ and *H*_FE_ − *H*_S_ in the sighted confirmed significant activation in the left IFG and left MTG during visual and haptic recognition of facial expressions (Table 2 and Figure 5, yellow area). The peak coordinates in the left IFG had a 20% probability of being located within area 44 and a 40% probability of being located in area 45 (Amunts et al., 1999).

**Figure 6:**
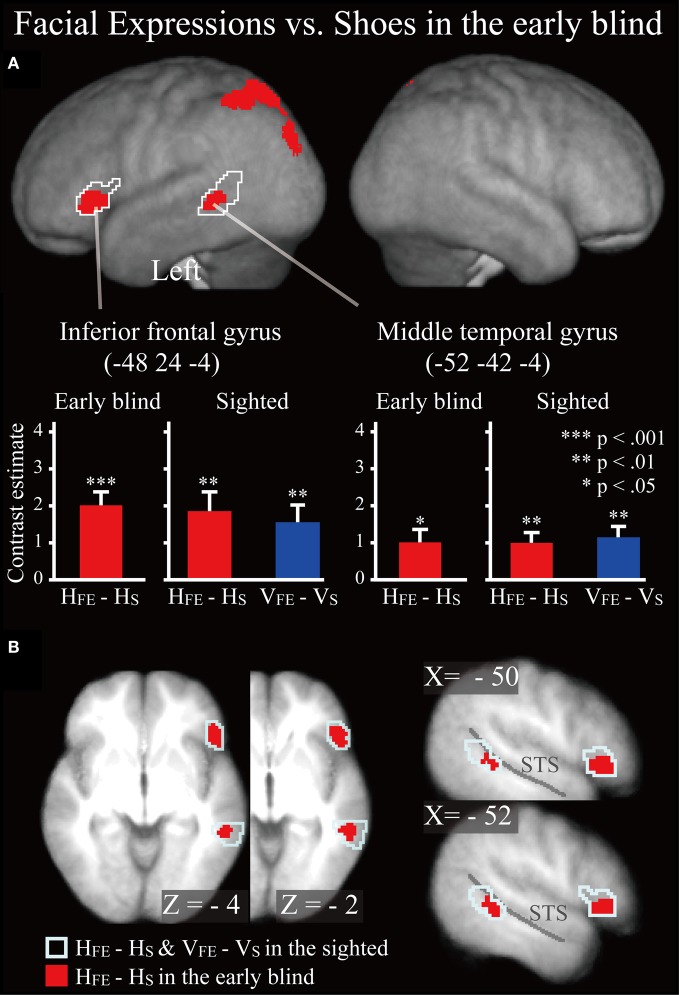
**SPM of the average neural activity within the early blind group during haptic identification of facial expressions compared with shoes (*H*_FE_ − *H*_S_). (A)** The activation patterns were superimposed on a surface-rendered T1-weighted high-resolution MRI averaged across the subjects. The white lines indicate regions activated during haptic and visual recognition in the sighted group (see Figure 5B). The bar graphs indicate the contrast estimates (i.e., activity) for identification of facial expressions relative to shoes using a volume of interest with a sphere of 8-mm diameter (corresponding to the size of the spatial-smoothing kernel applied to these data). Asterisks indicate the results of one-sample *t*-tests. Data are presented as the mean ± SEM of 17 early blind subjects. **(B)** The activation patterns were superimposed on the sagittal and transverse sections. The blue lines indicate regions activated during haptic and visual recognition in the sighted group.

***Early blind group.*** The contrast for haptic identification of facial expressions vs. shoes (*H*_FE_ − *H*_S_) in the early blind group activated regions within the left IFG and left MTG that were activated in the sighted group (Figure 6, regions within the white lines). The peak coordinates in the left IFG had a 10% probability of being located within area 44 and a 30% probability of being located in area 45 (Amunts et al., 1999). Moreover, this contrast also revealed a network of other brain regions, including the left superior parietal lobule, left superior occipital gyrus, and left middle occipital gyrus (Table 3).

**Table 3:** **Group analyses of brain regions in the early blind subjects**.

**Spatial extent test**		**MNI coordinate**			***Z*-value**	**Hemisphere**	**Anatomical region**
**Cluster size (mm^3^)**	***P*-values**	***x***	***y***	***x***			
528	<0.05^*^	−52	−42	−4	3.22	L	Middle temporal gyrus
840	<0.01^*^	−48	24	−4	3.64	L	Inferior frontal gyrus^a^
10008	<0.001	−16	−70	50	4.14	L	Superior parietal lobule
		−16	−88	32	4.02	L	Superior occipital gyrus
		−28	−84	40	2.66	L	Middle occipital gyrus

*The threshold size of activation was P < 0.05, corrected for multiple comparisons, when the height threshold was set at Z > 2.58; x, y, and z are stereotaxic coordinates (mm). Hem, Hemisphere; L, left*.

* *The search volume for activation was limited to each region activated by the sighted group*.

a *Probability value on cytoarchitectonic map: 10% for area 44 and 30% for area 45*.

**Figure 7:**
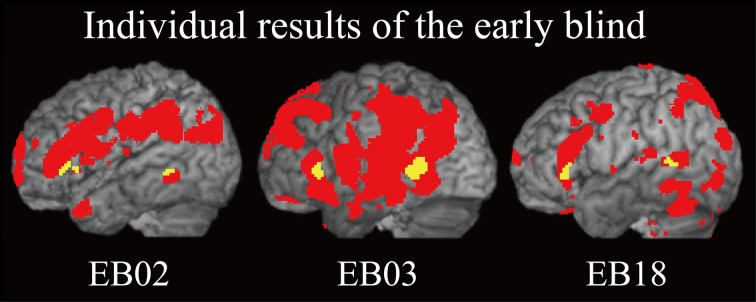
**Individual analysis on brain regions activated by identification of facial expressions (relative to that of shoes) in the early blind group.** The activation patterns during identification of FEEs relative to shoes were superimposed on a surface-rendered T1-weighted high-resolution MRI of each individual. The yellow-colored regions indicate overlap with regions of activation in the sighted group (Figure 5B, yellow-colored regions). Note that all of the three individuals were totally blind from birth. The height threshold was set at *Z* > 2.58, uncorrected for multiple comparisons.

***Does age at onset of total blindness affect activity in the IFG and MTG?*** We tested whether age at onset of total blindness in the early blind subjects correlated with activity in the left IFG and MTG. The former parameter did not significantly correlate with the contrast estimate (i.e., activity of facial expressions relative to that of shoe) in either the IFG or MTG (absolute *r* values <0.2). Moreover, one-sample *t*-tests on the contrast estimate confirmed that activity in each of the IFG and MTG was significantly greater than zero, regardless of whether or not the data for the four early blind subjects who became totally blind after birth were eliminated from the analyses (*P* values <0.05). Figure 7 shows the results of three congenitally blind individuals exhibiting activation in the IFG and MTG during haptic perception of facial expressions.

**Figure 8:**
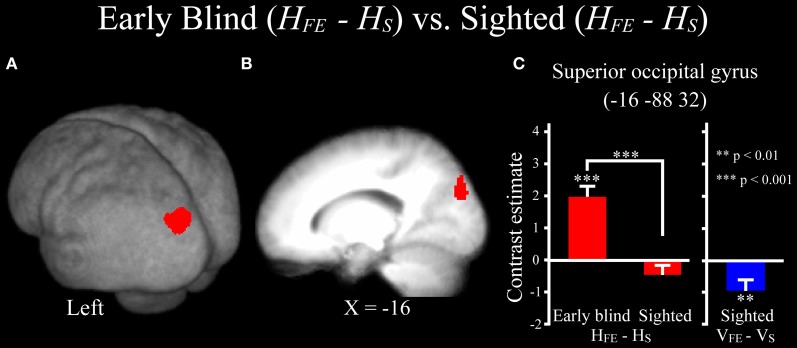
**Brain regions more strongly activated by the haptic identification of facial expressions relative to shoes (*H*_FE_ − *H*_S_) for the early blind compared with the sighted group.** This contrast was evaluated within the brain regions activated by *H*_FE_ − *H*_S_ in the early blind group. The size of activation was thresholded at *P* < 0.05, corrected for multiple comparisons, when the height threshold was set at *Z* > 2.58. **(A)** The activation patterns were superimposed on a surface-rendered T1-weighted high-resolution MRI averaged across the subjects. **(B)** The activation pattern was superimposed on the sagittal section. **(C)** The bar graphs indicate the activity (contrast estimates) for identification of facial expressions relative to shoes using a volume of interest with an 8-mm diameter sphere. Asterisks above the error bars indicate the results of the one-sample *t*-test on the contrast estimates, whereas asterisks between the error bars indicate the results of the independent *t*-tests. Data are presented as the mean ± SEM of 17 early blind and 22 sighted subjects.

***Differences in activation patterns for the *H*_FE_ − *H*_S_ contrast between the early blind and sighted groups.*** We examined which regions were activated more by the haptic identification of facial expressions (relative to shoes, i.e., the *H*_FE_ − *H*_S_ contrast) in the early blind relative to the sighted group. The contrast of *H*_FE_ − *H*_S_ in the blind group vs. *H*_FE_ − *H*_S_ in the sighted group, which was conducted within the brain regions activated by the *H*_FE_ − *H*_S_ contrast in the blind group, revealed significant activation in the left superior and middle occipital gyri (Figure 8 and Table 4). In this region, activation in response to the haptic identification of facial expressions was significantly greater than activation in response to shoes for the early blind subjects, whereas such activation was not observed for the sighted group (Figure 8). Similarly, we examined the brain regions activated more by the haptic identification of facial expressions (relative to shoes; the *H*_FE_ − *H*_S_ contrast) in the sighted group than in the early blind group, and discovered significant activation in the left cerebellum (Table 4).

**Table 4:** **Group comparisons of brain regions activated by haptic identification of facial expressions relative to shoes (*H*_FE_ − *H*_S_)**.

**Spatial extent test**		**MNI coordinate**			***Z*-value**	**Hemisphere**	**Anatomical region**
**Cluster size (mm^3^)**	***P*-values**	***x***	***y***	***z***			
**EARLY BLIND VS. SIGHTED CONTROL GROUP**							
1512	<0.05	−16	−88	32	4.08	L	Superior occipital gyrus
		−28	−84	40	2.66	L	Middle occipital gyrus
**SIGHTED CONTROL VS. EARLY BLIND GROUP**							
3688	<0.01	−2	−44	−20	4.26	L	Cerebellum

*The threshold size of activation was P < 0.05, corrected for multiple comparisons, when the height threshold was set at Z > 2.58; x, y, and z are stereotaxic coordinates (mm). Hem, Hemisphere; L, left*.

## Discussion

### Behavioral performance

In the psychophysical experiment, early blind and sighted groups identified basic facial expressions using touch alone at levels well above chance. Moreover, the patterns of behavioral performance by these two groups were highly similar. As no corrective feedback was provided during either training or experimental trials in this experiment, it is unlikely that subjects had learned the correct associations between facemasks and labels of facial expression during the experiment. Collectively, our behavioral data support the hypothesis that the recognition of basic facial expressions can be mediated by a supramodal representation, regardless of visual experience.

### Neural representation underlying the recognition of facial expressions regardless of visual experience

We observed activation of the IFG and MTG during haptic and visual identification of facial expressions in the sighted. Moreover, such activity was also observed during the recognition of facial expressions in the early blind. Previous neuroimaging studies have shown that the IFG and pSTS region (including the MTG) in sighted individuals are involved in the recognition of facial expressions. These areas are engaged whether the modality is visual (Gorno-Tempini et al., 2001; Narumoto et al., 2001; Carr et al., 2003; Montgomery and Haxby, 2008) or haptic (Kitada et al., 2010). The present study extends these findings by revealing for the first time that the IFG and MTG in early blind individuals—not just the sighted—are activated by the recognition of facial expressions. This result indicates that the neural systems underlying the recognition of basic facial expressions can develop supramodally *even* in the absence of early visual experience.

It is known that early visual deprivation results in plastic change in early visual areas such as the striate cortex (Sadato et al., 1996; Cohen et al., 1997). For instance, transcranial magnetic stimulation (TMS) over the early visual cortices reduced performance accuracy of Braille reading in early blind subjects (Cohen et al., 1997). By contrast, an increasing number of neuroimaging studies have shown that the functional organization of other brain regions is highly similar, regardless of visual experience: the ventral visual pathway (Amedi et al., 2007; Mahon et al., 2009; Reich et al., 2011; Wolbers et al., 2011; Striem-Amit et al., 2012), dorsal visual pathway (Poirier et al., 2006; Ricciardi et al., 2007; Matteau et al., 2010), limbic areas (Klinge et al., 2010), and the action-understanding network (Ricciardi et al., 2009). Our result extends these findings by demonstrating that cortical areas beyond the early visual cortex are functionally organized in a supramodal fashion for representing facial expressions.

There are two possible accounts for the role played by the IFG and MTG in the recognition of facial expressions. First, these brain regions might be related to cognitive processing of different aspects of faces including facial expressions. For instance, Tsao et al. (2008a,b) demonstrated that the ventral prefrontal cortex and the superior temporal sulcus in the non-human primates contain regions (patches) selectively active during observation of faces. In human neuroimaging studies, a number of studies have revealed that observation of faces (relative to the other categories of common objects) selectively activates regions in and around the superior temporal sulcus (e.g., Puce et al., 1996; Haxby et al., 1999). Therefore, it is possible that the IFG and MTG, which are involved in the processing of face perception, may contribute to the formation of representations that underlie the recognition of basic facial expressions, even in the absence of visual experience.

Alternatively, the IFG and MTG might be related to understanding the facial actions of others. It is well known that the execution and recognition of actions share common neural representations (“action representations”) (Prinz, 1992; Rizzolatti et al., 2001; Iacoboni and Dapretto, 2006; Jeannerod, 2006). Facial expressions can be considered as one such action class, because facial expressions can be decomposed into “action patterns” that involve the contraction and relaxation of sets of muscles (Ekman and Friesen, 1978). The notion of a common neural representation indicates that in order to understand the action of another person, that action must be motorically simulated by the perceiver. As supporting evidence, electromyographic studies have previously shown that muscles involved in creating facial expressions are also activated when a perceiver merely observes others portraying expressions (Dimberg and Thunberg, 1998; Dimberg et al., 2000). In addition, the IFG and pSTS region are activated not only visual observation, but also by visual imitation of static facial expressions (Carr et al., 2003; Montgomery and Haxby, 2008). As visual observation and imitation are unavailable to early blind individuals, they find it difficult to consciously control facial muscles accurately (Rinn, 1991; Galati et al., 1997). However, early blind individuals spontaneously produce facial expressions of basic emotions that are similar to those produced by sighted individuals (Thompson, 1941; Darwin, 1998; Galati et al., 2001, 2003; Matsumoto and Willingham, 2009). For instance, Galati et al. (2001) demonstrated that congenitally blind and sighted children produce highly similar facial expressions in situations which arouse basic emotions (i.e., anger, joy, disgust, surprise, sadness, and fear). Therefore, it is possible that early blind individuals haptically recognize the facial expressions of others by mentally and motorically simulating the facial expressions that they spontaneously produce. Further studies are necessary to specify the cognitive function of the IFG and MTG during the recognition of facial expressions.

Development of the representation of basic facial expressions can be influenced by both pre-natal and post-natal factors. Previous developmental studies have shown that representation of basic facial expressions appears to emerge very early in life. For instance, even neonates can visually imitate and discriminate among basic facial expressions (Field et al., 1982). Moreover, the observation of female faces (relative to the observation of lights of diodes) activates brain regions including the IFG and MTG (Tzourio-Mazoyer et al., 2002). Coupled with the findings from the present study, it is possible that the IFG and MTG might be innately organized for the computations necessary for representing faces including basic facial expressions.

At the same time, sensory cues from non-visual modalities should contribute to forming representations of facial expressions in these brain regions because sensory experience is typically required for an innate mechanism to refine and develop itself toward a more mature form (Greenough et al., 1987; Leppänen and Nelson, 2009). For instance, kinesthetic feedback from spontaneously produced facial expressions could trigger and enhance the development of representations of facial expressions in the absence of vision. This possibility is consistent with the finding that infant monkeys maintained their preference for visually presented faces over other objects after being deprived of face-related visual stimuli for 2 years after birth (Sugita, 2008). Sugita interpreted this finding that face representations could be formed through non-visual sensory modalities, such as kinesthetic feedback from self-produced facial movements. It is possible that action representations in early blind individuals may utilize such kinesthetic feedback as prior knowledge in order to interpret the facial expressions of others.

In the present study, the left hemisphere was mainly activated during haptic identification of facial expressions, and is thus consistent with our previous finding (Kitada et al., 2010). It is unlikely that the left-lateralized activation is merely the result of naming facial expressions because this component was factored out in our analyses using the control condition (the identification of shoes). Alternatively, it is possible that manual exploration with the right hand enhanced activation of the contralateral (left) hemisphere relative to the ipsilateral (right) hemisphere. Although the right hemisphere is typically dominant in face perception, the IFG and MTG in the left hemisphere may also play a critical role. For instance, activation of the IFG and MTG in 2-month-old infants was greater in the left hemisphere than the right hemisphere during visual observation of face (Tzourio-Mazoyer et al., 2002). Pourtois et al. (2005) utilized a repetitive suppression paradigm to specify brain regions involved in the recognition of familiar faces. The authors found that a repetition-suppression effect was specifically found in the left IFG and MTG. Moreover, the left-sided activation can be explained in terms pertaining to the action-understanding network, since visual and auditory observation of hand actions commonly activates brain regions, including the IFG and MTG in the left, but not the right, hemisphere (Gazzola et al., 2006). Thus, it is possible that the left IFG and MTG play a critical role in analyzing the actions of others based on sensory cues from multiple modalities.

In sum, our findings suggest that a common neural representation underlying the recognition of facial expressions is present, regardless of visual experience. We propose that both innate neural mechanisms and non-visual sensory cues (e.g., kinesthesis) may contribute to the development of such supramodal representation.

### Neural representation specific for touch

The superior parietal lobule was also activated during haptic face identification, regardless of subject group; however, this region was not activated by vision, as found previously with sighted subjects (Kitada et al., 2010). Previous neuroimaging studies have revealed that this region is involved in haptic spatial processing of objects (Sathian et al., 1997; Kaas et al., 2007; Stilla and Sathian, 2008). Our stimuli required extensive serial haptic exploration. Moreover, unlike shoes, facemasks are rarely haptically explored (if at all) in daily life. Accordingly, haptic face identification might impose greater demands for spatial integration in this brain region (relative to shoes) than does corresponding visual identification, since both object classes are not only highly familiar but also more efficiently processed on the basis of simultaneous visual inputs.

The superior and middle occipital gyri were activated during haptic identification of facial expressions (relative to shoes) in early blind subjects; however neither visual nor haptic perception of facial expressions activated this region in the sighted subjects. This result suggests that the brain network underlying haptic face recognition might differ between early blind and sighted groups. Previous studies have suggested that the occipital cortex is critical for the tactile processing of spatial patterns in early blind individuals (Sadato et al., 1996; Cohen et al., 1997; Pietrini et al., 2004; Stilla et al., 2008; Fujii et al., 2009). Early visual deprivation may result in plastic change in these regions such that they become more sensitive to the spatial properties of haptic inputs.

### Future application

It is known that early visual deprivation can delay the development of social cognition (Keeler, 1958; Wing, 1969; Brown et al., 1997; Hobson et al., 1999; Hobson and Lee, 2010). Several behavioral studies have shown that early visual deprivation reduces voluntary control of facial expressions, a limitation that extends into adulthood (Webb, 1977; Marshall and Peck, 1986; Rinn, 1991; Galati et al., 1997). For future consideration, we propose that haptic recognition of a caregiver's facial expressions may potentially serve as a valuable substitute for visual recognition as a means by which early blind individuals may reduce, if not overcome, current difficulty in producing facial expressions voluntarily. Such a haptic approach to intervention may further facilitate the development of social cognition, which is typically mediated by vision.

## Conclusion

The present study demonstrates that humans can haptically identify basic facial expressions, regardless of early visual experience. Haptic identification of facial expressions by both the early blind and sighted groups activated regions in the IFG and MTG, which were similarly activated by the visual identification of facial expressions (relative to shoes). Hence, despite early visual deprivation, humans are still capable of neurally representing facial expressions. We conclude that the brain network that subserves haptic recognition of facial expressions can develop effectively without the benefits of early visual experience.

### Conflict of interest statement

The authors declare that the research was conducted in the absence of any commercial or financial relationships that could be construed as a potential conflict of interest.
